# Cross-Sectional Study of Flood Damage Assumptions in Medical Facilities Using Geographic Information Systems

**DOI:** 10.7759/cureus.59577

**Published:** 2024-05-03

**Authors:** Yuji Kaneko, Hideo Komine, Yuki Kataoka

**Affiliations:** 1 Department of Medicine, Hokkaido University, Sapporo, JPN; 2 Department of Civil & Environmental Engineering, Waseda University, Tokyo, JPN; 3 Section of Clinical Epidemiology, Department of Community Medicine, Kyoto University Graduate School of Medicine, Kyoto, JPN; 4 Department of Healthcare Epidemiology, Kyoto University Graduate School of Medicine/School of Public Health, Kyoto, JPN; 5 Department of Systematic Reviewers, Scientific Research Works Peer Support Group, Osaka, JPN; 6 Department of Internal Medicine, Kyoto Min-iren Asukai Hospital, Kyoto, JPN

**Keywords:** business continuity plan, disaster base hospital, damage, flood, geographic information system, cross-sectional study

## Abstract

Introduction

Floods not only directly damage medical facilities but also hinder access to medical facilities, potentially disrupting local medical services. The scale of damage that medical facilities suffer from floods in Japan is unknown. In this study, we assessed the potential impact of floods on Japanese healthcare facilities by facility characteristics.

Methods

We conducted a cross-sectional study involving medical facilities registered in the Japan Medical Association Regional Medical Information System. Geographic data for the inundation area was obtained from open data of the Japanese government. Facilities that overlap with flooded areas were designated as affected facilities. The primary outcomes were the percentage of damaged facilities and beds. We calculated odds ratios (OR) and 95% confidence intervals (95%CI) using the Wald method to assess the impact of disaster base hospital designation on damage extent.

Results

We included 140,826 general clinics and 8,126 hospitals, which had 137,731 and 1,483,347 beds, respectively. The planned scale of flooding is estimated to affect 8.0% of general clinics and 10.8% of their beds. For hospitals, these figures were 8.8% and 7.8%, respectively. The maximum potential scale of flooding is estimated to affect 23.6% of general clinics and 23.9% of their beds. For hospitals, these figures were 22.5% and 20.6%, respectively. At the planned scale of flooding, there was no difference found in the rate of damaged facilities between disaster base hospitals and non-disaster base hospitals, and the rate of damaged beds was lower at non-disaster base hospitals (OR = 0.92, 95%CI = 0.71-1.18 for damaged facilities and OR = 0.79, 95%CI = 0.78-0.80 for damaged beds). At the maximum potential scale of flooding, there was no difference found in the expected damage between disaster base hospitals and non-disaster base hospitals (OR = 1.14, 95%CI = 0.95-1.38 for damaged facilities and OR = 0.99, 95%CI = 0.98-1.00 for damaged beds).

Conclusion

In Japan, floods can hinder nationwide medical functions, particularly in certain regions. Healthcare professionals should assess potential flood damage in advance and ensure that their workplace's business continuity plan includes appropriate countermeasures.

## Introduction

Floods occur when rivers and lakes overflow their banks due to heavy rainfall and other factors [1]. Global warming has contributed to an increase in floods worldwide [2]. The World Meteorological Organization reports that the 2010s saw approximately six times more floods than the 1970s, resulting in about 600,000 deaths [3]. In recent years, Japan has experienced extensive flood damage such as the torrential rains that struck western Japan in 2018 and Kyushu region in July 2020. Floods not only directly damage medical facilities but also hinder access to medical facilities [4,5], potentially disrupting local medical services.

To enhance resilient disaster prevention and mitigation capabilities in Japan, the Cabinet Office formulated the Strategic Innovation Program Research and Development Plan (referred to as SIP plan) in April 2018 [6]. SIP plan underscores disaster forecasting as a critical component of resilience, defined as the ability to minimize damage, recover swiftly, and return to normal life. SIP plan sets a direction for technology-mediated disaster research and emphasizes government-led initiatives in disaster medicine. Specifically, the plan advocates for the use of geographic information systems (referred to as GIS). A cross-sectional study clarified the activity policy and optimal dispatch of medical support teams, such as Disaster Medical Assistance Teams (DMATs), using GIS data [7].

Cooperation between disaster base hospitals and primary care facilities is important to meet the rapidly increasing demand for medical care after disasters. Disaster base hospitals are key medical facilities in times of disaster [8]. They are expected to maintain and strengthen local medical functions and provide prompt and appropriate medical care to disaster victims in Japan. After the disaster, both long-term evacuation and home evacuation increase chronic diseases and exacerbation of frailty [9,10]. Therefore, the function of primary care will become even more important. These medical facilities are expected to resume functioning without delay and meet medical needs at the time of the disaster through smooth cooperation.

Few studies have estimated the potential damage to medical facilities from floods. A cross-sectional study showed the extent of flood-related damage in hospitals with infectious disease beds [11]. To the best of our knowledge, comprehensive studies on primary care facilities, including disaster base hospitals and general clinics, are lacking. Furthermore, no studies addressed the distance between medical facilities and rivers.

In this study, we assessed the potential impact of floods on medical facilities in Japan and determined if designated disaster base hospitals experienced different levels of damage. In addition, we evaluated the distance of medical facilities to rivers.

## Materials and methods

Study design

This is a cross-sectional study using open data.

Study subject

We included medical facilities, excluding dental practices, registered in the Japan Medical Association Regional Medical Information System (JMAP) as of November 2022 [12]. Medical facilities are mainly divided into general clinics and hospitals. General clinics are further subdivided into internal medicine, surgery, pediatrics, obstetrics and gynecology, dermatology, otolaryngology, ophthalmology, and psychiatry. In Japan, these clinics may be responsible for primary care in the community. General clinics specializing in multiple departments were counted for each specialty. We categorized hospitals as either disaster base hospitals or non-disaster base hospitals. Disaster base hospitals are designated by the Ministry of Health, Labor and Welfare (referred to as MHLW) as of April 2023 [13]. The included medical facilities represent approximately 100% of all medical facilities providing insured care in Japan.

Data collection method

We obtained geographic river data and data for inundation areas due to floods from the flood inundation area data (river unit) provided by the Ministry of Land, Infrastructure, Transport and Tourism (MLIT) [14], as of October 28, 2023. River data is the curve information connecting centerlines of river flow paths, as defined by the River Law. We used two types of data for inundation areas from this source.

Planned Scale of Flooding

The planned scale of flooding is defined as areas expected to be inundated by a river overflow as a result of rainfall occurring once every 10-200 years. This definition aligns with the basic policy for river improvement under the River Law. This indicator is used as a standard for planning flood prevention measures, including river rehabilitation.

Maximum Potential Scale of Flooding

The maximum potential scale of flooding is defined as areas expected to be inundated by a river overflow as a result of rainfall occurring once every 1,000 years.

Analysis method

This study employed GIS to manage, process, and analyze spatial data based on geographic location [15]. GIS data encompasses elements such as cities, rivers, roads, and buildings, along with map images that include these features.

In this study, we used GeoPandas ver. 0.13.2 [16], a Python library, to analyze GIS data. We plotted the coordinates of inundation areas and medical facilities on a map of Japan. We then recorded the number of facilities and hospital beds at risk of flooding. We categorized medical facilities into general clinics and hospitals. All inundated areas had the same risk.

We classified hospitals into "disaster base hospitals" and "non-disaster base hospitals" according to the MHLW approval status as of April 2023. We identified facilities as damaged if they overlapped with flood-affected areas in the GIS for medical facilities.

We estimated the damage based on the percentage of damaged facilities and beds. We also visualized damage estimates for each prefecture on a heat map, in addition to a nationwide assessment for Japan. We calculated the odds ratio (OR) and 95% confidence interval (95%CI) using the Wald method to assess whether the degree of damage differs depending on whether the hospital is designated as a disaster center or not.

We examined the distance to the nearest river for facilities. The analysis focused on internal medicine facilities, which comprise the largest number of general clinics, and hospitals. We generated a river centerline curve from location data and mapped it with the relevant medical facilities. We then used the longitude and latitude to calculate the distance to the nearest river from each medical facility. We used GeoPy ver. 2.3.0 [17], a Python library, to find and locate the coordinates of addresses around the world and calculate the distance.

Ethical considerations

This study relied solely on open data and did not involve personal information.

## Results

Nationwide damage estimates

We included 140,826 general clinics and 8,126 hospitals, which had 137,731 and 1,483,347 beds, respectively (Table 1).

**Table 1:** Basic characteristics of the surveyed medical facilities and beds

Medical facilities	Medical facilities, n	Medical beds, n
General clinic	140,826	137,731
Internal medicine	56,823	46,213
Surgery	24,180	35,048
Pediatrics	21,317	16,527
Obstetrics and gynecology	4,766	21,077
Dermatology	12,584	7,643
Otorhinolaryngology	5,718	1,811
Ophthalmology	8,079	6,262
Psychiatry	7,359	3,150
Hospital	8,126	1,483,347
Disaster base hospital	770	352,422
Non-disaster base hospital	7,356	1,130,925

The planned scale of flooding is estimated to affect 8.0% of general clinics and 10.8% of their beds. For hospitals, these figures were 8.8% and 7.8%, respectively. The maximum potential scale of flooding is estimated to affect 23.6% of general clinics and 23.9% of their beds. For hospitals, these figures were 22.5% and 20.6%, respectively (Table 2).

**Table 2:** Predicting flood damage in medical facilities nationwide

Medical facilities	Planned scale of flooding		Maximum potential scale of flooding	
	Damaged facilities (%)	Damaged beds (%)	Damaged facilities (%)	Damaged beds (%)
General clinic	8.0	10.8	23.6	23.9
Internal medicine	7.7	10.9	22.9	23.3
Surgery	7.8	10.8	23.3	23.4
Pediatrics	8.9	11.4	23.7	24.8
Obstetrics and gynecology	7.8	10.4	23.8	26.0
Dermatology	8.1	9.8	24.6	23.2
Otorhinolaryngology	8.4	11.4	25.6	28.2
Ophthalmology	8.0	10.7	25.5	23.0
Psychiatry	7.5	11.2	24.8	20.2
Hospital	8.8	7.8	22.5	20.6
Disaster base hospital	9.5	9.2	20.4	20.7
Non-disaster base hospital	8.8	7.4	22.7	20.6

Among the 770 disaster base hospitals with 352,422 beds, 9.5% of facilities and 9.2% of beds were expected to be damaged at the planned scale of flooding. At the maximum potential scale of flooding, these figures were expected to be 20.4% and 20.7%, respectively. In contrast, among the 7,356 non-disaster base hospitals with 1,130,925 beds, 8.8% of facilities and 7.4% of beds were expected to be damaged at the planned scale of flooding. At the maximum potential scale of flooding, these figures were expected to be 22.7% and 20.6%, respectively (Table 2).

Expected scale of damage by prefecture

**Figure 1:**
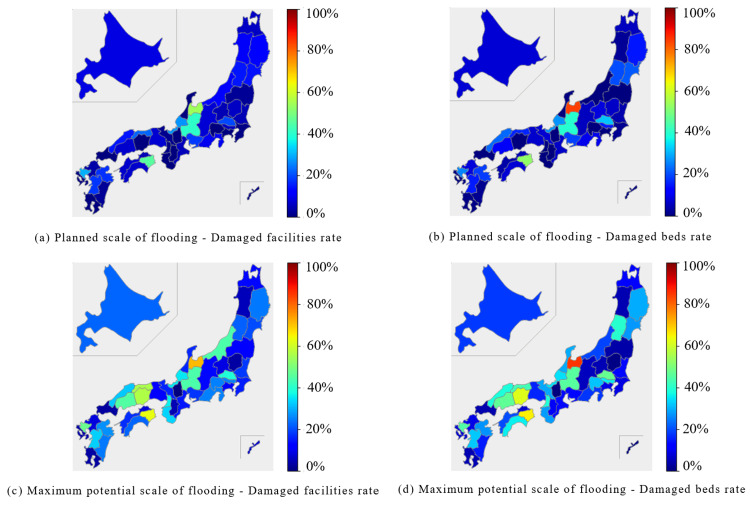
Percentage of damaged facilities and beds for "Internal Medicine" expected to be affected by the planned scale and maximum potential scale of flooding are mapped for each prefecture The percentage of damaged facilities was calculated by dividing the number of damaged facilities by the number of total facilities. (a) Planned scale of flooding - Damaged facilities rate. (b) Planned scale of flooding - Damaged beds rate. (c) Maximum potential scale of flooding - Damaged facilities rate. (d) Maximum potential scale of flooding - Damaged beds rate

**Figure 2:**
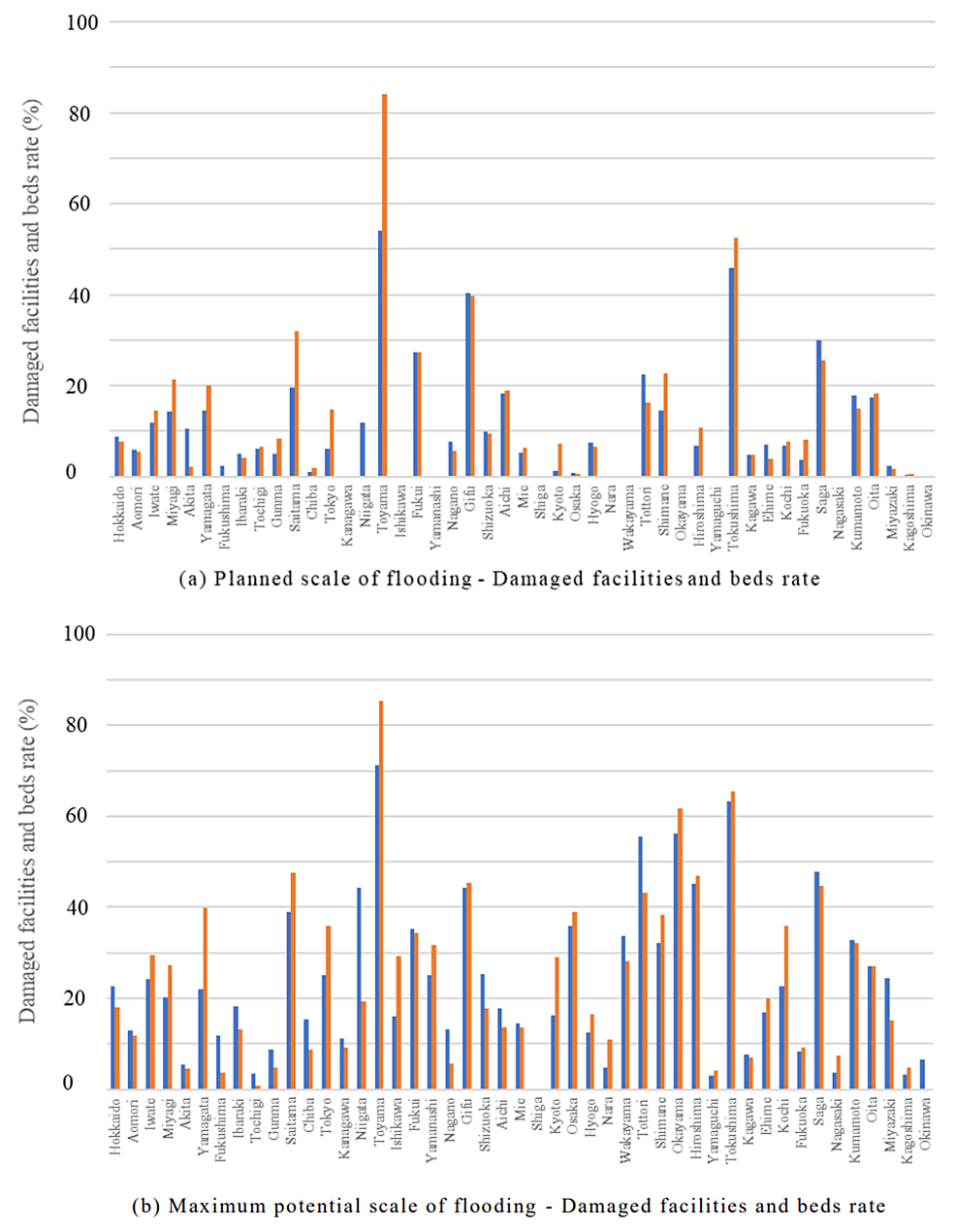
Damage rates for "Internal Medicine" in each of the 47 prefectures Blue bars represent the percentage of damaged facilities, and orange bars represent the percentage of damaged beds. (a) Planned scale of flooding - Damaged facilities and beds rate. (b) Maximum potential scale of flooding - Damaged facilities and beds rate

In the internal medicine category, the Chubu region and parts of the Shikoku region were predicted to experience extensive damage during the planned scale of flooding. In the Chubu region, both the percentage of damaged facilities and beds were expected to exceed 40%. In the Shikoku region, the Tokushima prefecture reported approximately 50% of facilities and beds were expected to be damaged. At the maximum potential scale of flooding, the affected facilities and beds were expected to exceed 50% in the Chubu, Chugoku, and Shikoku regions (Figure 1, Figure 2).

**Figure 3:**
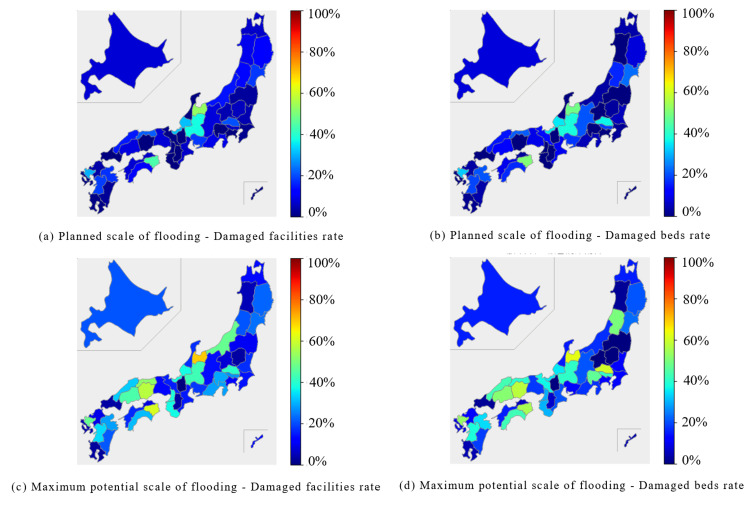
Percentage of damaged facilities and beds for "Surgery" expected to be affected by the planned scale and maximum potential scale of flooding are mapped for each prefecture The percentage of damaged facilities was calculated by dividing the number of damaged facilities by the number of total facilities. (a) Planned scale of flooding - Damaged facilities rate. (b) Planned scale of flooding - Damaged beds rate. (c) Maximum potential scale of flooding - Damaged facilities rate. (d) Maximum potential scale of flooding - Damaged beds rate

In the surgical category, the Chubu region and parts of the Shikoku region were predicted to experience extensive damage during the planned scale of flooding, with over 40% of facilities and beds affected in some areas. The Tokushima prefecture was expected to experience approximately 50% damage to both facilities and beds. At the maximum potential scale of flooding, the Chubu, Chugoku, Shikoku, and Kyushu regions saw over 50% of facilities and beds were predicted to be damaged. Notably, Niigata and Yamagata prefectures, along with parts of the Kanto region, were expected to exhibit variability in the extent of damage to facilities and beds (Figure 3).

**Figure 4:**
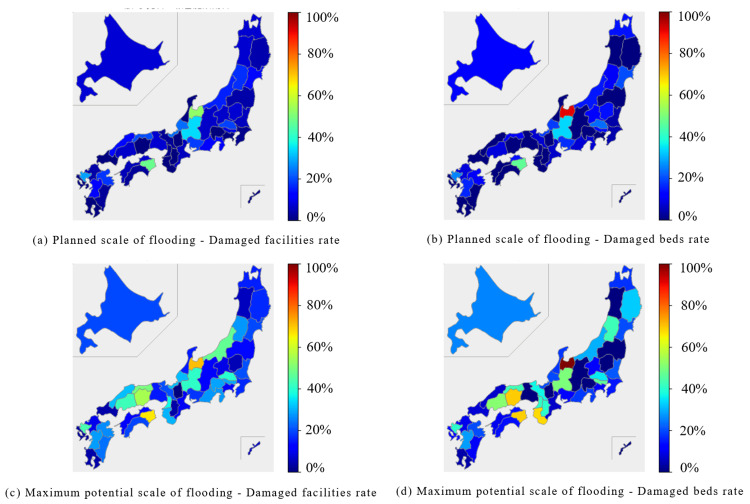
Percentage of damaged facilities and beds for "Pediatrics" expected to be affected by the planned scale and maximum potential scale of flooding are mapped for each prefecture The percentage of damaged facilities was calculated by dividing the number of damaged facilities by the number of total facilities. (a) Planned scale of flooding - Damaged facilities ratio. (b) Planned scale of flooding - Damaged beds rate. (c) Maximum potential scale of flooding - Damaged facilities rate. (d) Maximum potential scale of flooding - Damaged beds rate

In the pediatrics category, the Chubu region and parts of the Shikoku region were predicted to experience damage to over 40% of facilities and beds during the planned scale of flooding. The Toyama prefecture was expected to suffer the greatest damage, with approximately 90% of beds affected. At the maximum potential scale of flooding, the Chubu, Kinki, Chugoku, and Shikoku regions were expected to have more than 60% of either their facilities or beds damaged (Figure 4).

**Figure 5:**
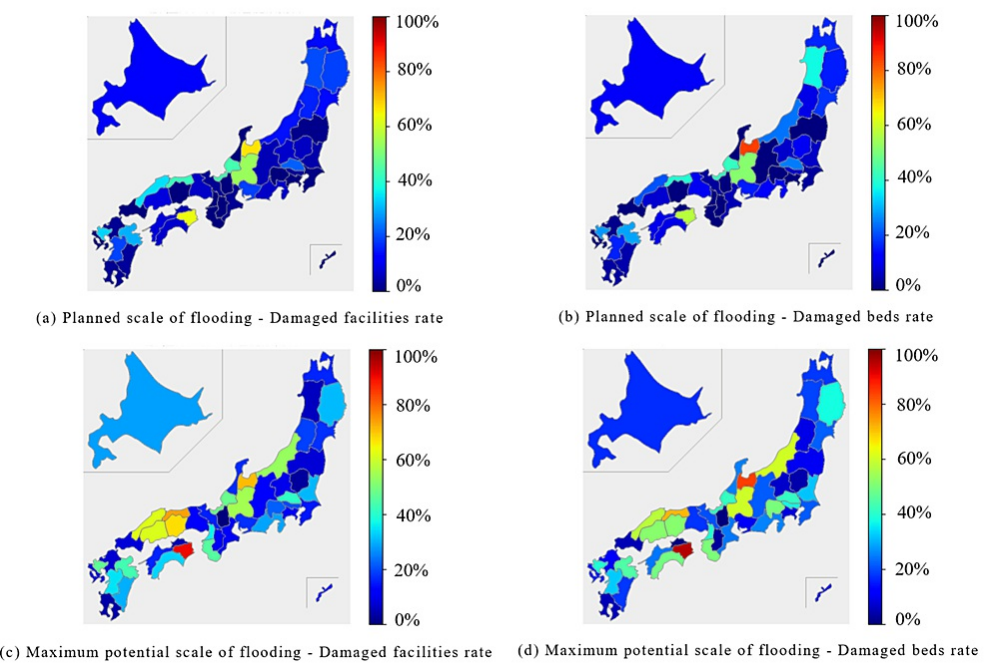
Percentage of damaged facilities and beds for "Obstetrics and Gynecology" expected to be affected by the planned scale and maximum potential scale of flooding are mapped for each prefecture The percentage of damaged facilities was calculated by dividing the number of damaged facilities by the number of total facilities. (a) Planned scale of flooding - Damaged facilities rate. (b) Planned scale of flooding - Damaged beds rate. (c) Maximum potential scale of flooding - Damaged facilities rate. (d) Maximum potential scale of flooding - Damaged beds rate

In the obstetrics and gynecology category, the Chubu region, the Sea of Japan side of the Chugoku region, and the Shikoku region were predicted to experience damage to over 40% of facilities and beds during the planned scale of flooding. At the maximum potential scale of flooding, the Hokuriku, Chubu, Chugoku, Shikoku, and Kyushu regions were predicted to experience damage to more than 50% of facilities and beds (Figure 5).

**Figure 6:**
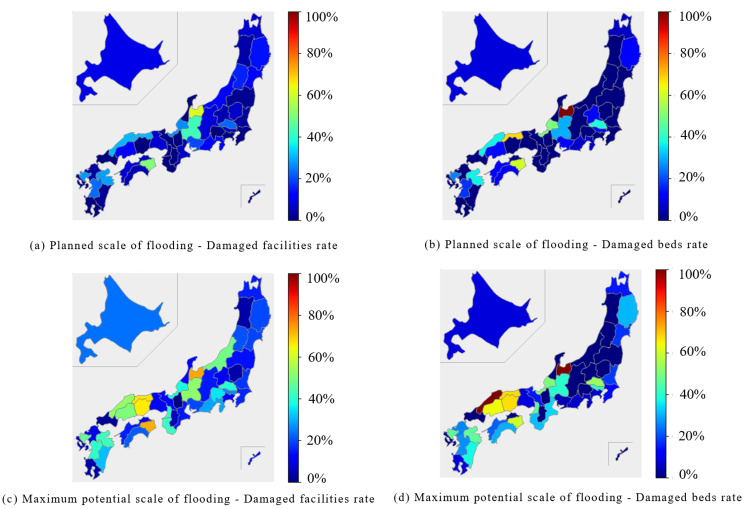
Percentage of damaged facilities and beds for "Dermatology" expected to be affected by the planned scale and maximum potential scale of flooding are mapped for each prefecture The percentage of damaged facilities was calculated by dividing the number of damaged facilities by the number of total facilities. (a) Planned scale of flooding - Damaged facilities rate. (b) Planned scale of flooding - Damaged beds rate. (c) Maximum potential scale of flooding - Damaged facilities rate. (d) Maximum potential scale of flooding - Damaged beds rate

In the dermatology category, the Chubu region, the Sea of Japan side of the Chugoku region, and the Shikoku region were predicted to experience damage to more than 50% of facilities or beds during the planned scale of flooding. At the maximum potential scale of flooding, the Hokuriku, Kanto, Chubu, Chugoku, Shikoku, and Kyushu regions were predicted to experience similar damage levels (Figure 6).

**Figure 7:**
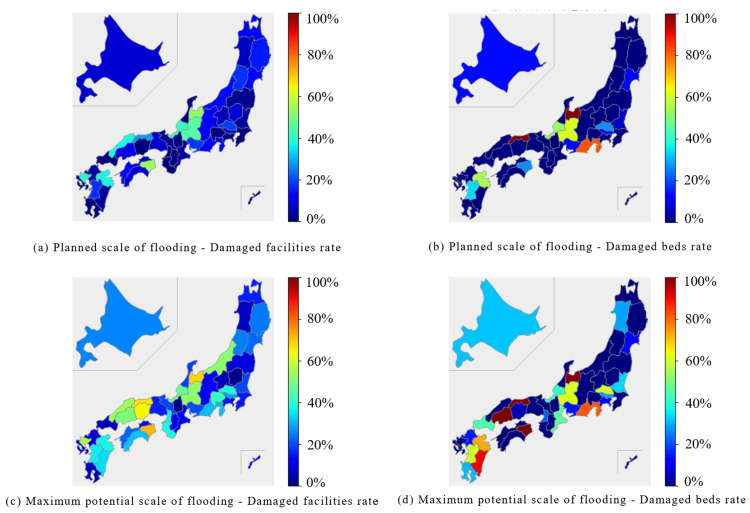
Percentage of damaged facilities and beds for "Otorhinolaryngology" expected to be affected by the planned scale and maximum potential scale of flooding are mapped for each prefecture The percentage of damaged facilities was calculated by dividing the number of damaged facilities by the number of total facilities. (a) Planned scale of flooding - Damaged facilities rate. (b) Planned scale of flooding - Damaged beds rate. (c) Maximum potential scale of flooding - Damaged facilities rate. (d) Maximum potential scale of flooding - Damaged beds rate

In the otorhinolaryngology category, the Chubu, Chugoku, Shikoku, and Kyushu regions were predicted to experience either the percentage of damaged facilities or beds exceeding 50% during the planned scale of flooding. Specifically, in the Toyama, Shizuoka, and Tottori prefectures, over 80% of beds were expected to be damaged. At the maximum potential scale of flooding, the Kanto, Chubu, Chugoku, Shikoku, and Kyushu regions were predicted to experience damage to more than 60% of either facilities or beds (Figure 7).

**Figure 8:**
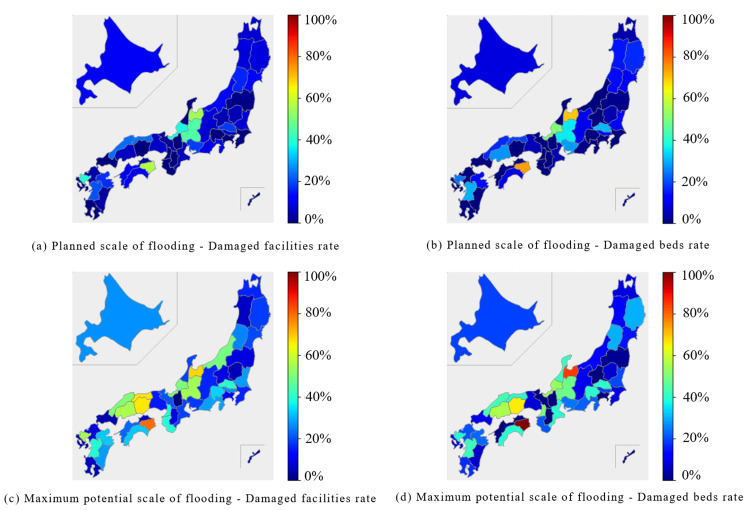
Percentage of damaged facilities and beds for "Ophthalmology" expected to be affected by the planned scale and maximum potential scale of flooding are mapped for each prefecture The percentage of damaged facilities was calculated by dividing the number of damaged facilities by the number of total facilities. (a) Planned scale of flooding - Damaged facilities rate. (b) Planned scale of flooding - Damaged beds rate. (c) Maximum potential scale of flooding - Damaged facilities rate. (d) Maximum potential scale of flooding - Damaged beds rate

In the ophthalmology category, the Chubu and Shikoku regions were predicted to experience damage to more than 50% of facilities and beds during the planned scale of flooding. At the maximum potential scale of flooding, the Chubu, Chugoku, and Shikoku regions were predicted to experience damage to over 60% of facilities or beds (Figure 8).

**Figure 9:**
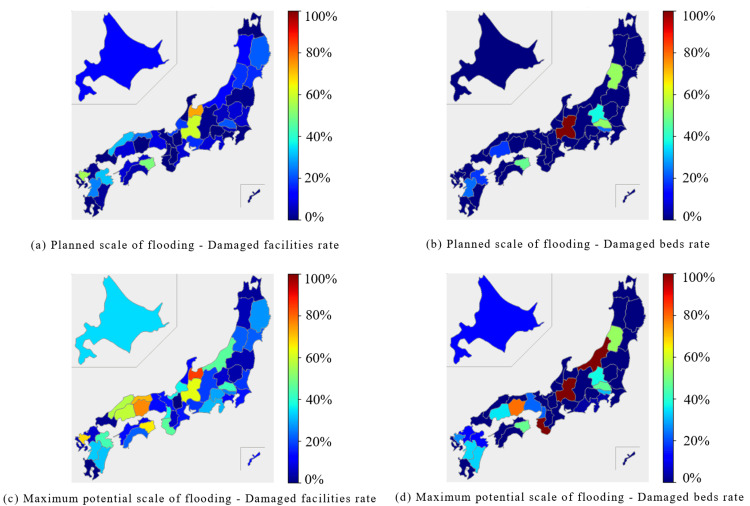
Percentage of damaged facilities and beds for "Psychiatry" expected to be affected by the planned scale and maximum potential scale of flooding are mapped for each prefecture The percentage of damaged facilities was calculated by dividing the number of damaged facilities by the number of total facilities. (a) Planned scale of flooding - Damaged facilities rate. (b) Planned scale of flooding - Damaged beds rate. (c) Maximum potential scale of flooding - Damaged facilities rate. (d) Maximum potential scale of flooding - Damaged beds rate

In the psychiatry category, the Tohoku, Kanto, Chubu, Shikoku, and Kyushu regions were predicted to experience damage to more than 60% of facilities and beds during the planned scale of flooding. At the maximum potential scale of flooding, the Tohoku, Hokuriku, Kinki, Chugoku, Shikoku, and Kyushu regions were predicted to experience damage to over 60% of facilities or beds (Figure 9).

**Figure 10:**
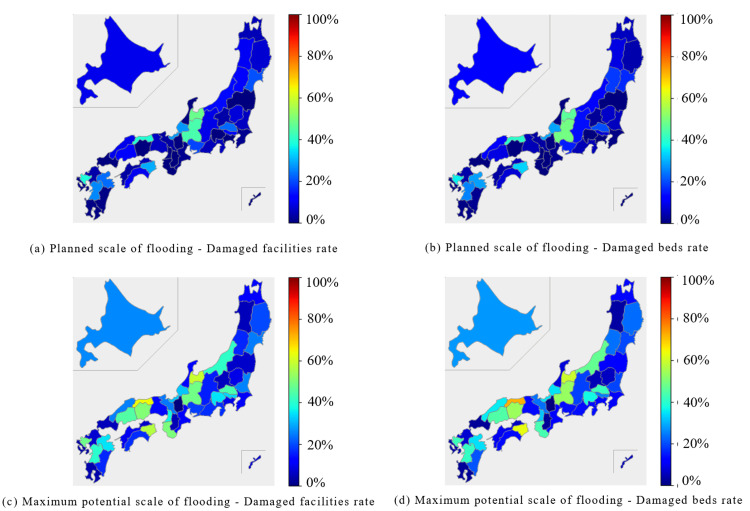
Percentage of damaged facilities and beds for "Hospital" expected to be affected by the planned scale and maximum potential scale of flooding are mapped for each prefecture The percentage of damaged facilities was calculated by dividing the number of damaged facilities by the number of total facilities. (a) Planned scale of flooding - Damaged facilities rate. (b) Planned scale of flooding - Damaged beds rate. (c) Maximum potential scale of flooding - Damaged facilities rate. (d) Maximum potential scale of flooding - Damaged beds rate

**Figure 11:**
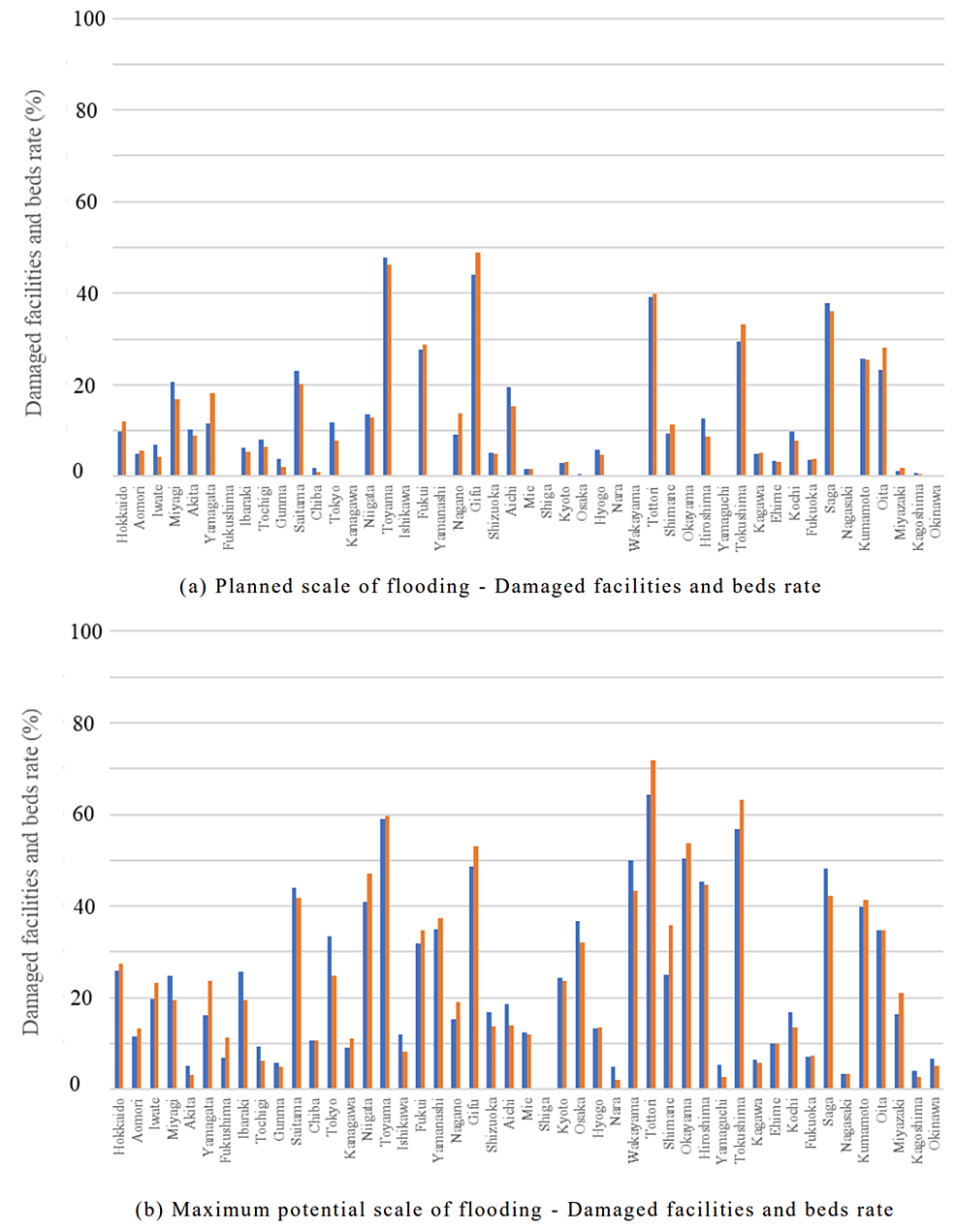
Damage rates for "Hospital" in each of the 47 prefectures Blue bars represent the percentage of damaged facilities, and orange bars represent the percentage of damaged beds. (a) Planned scale of flooding - Damaged facilities and beds rate. (b) Maximum potential scale of flooding - Damaged facilities and beds rate

In the hospital category, the Chubu region was predicted to experience extensive damage during the planned scale of flooding, with both the percentage of damaged facilities and beds exceeding 40% in some areas. At the maximum potential scale of flooding, the Hokuriku, Chubu, and Chugoku regions were expected to experience extensive damage, with several prefectures reporting over 40% of facilities and beds (Figure 10, Figure 11).

Comparing the extent of damage between disaster base hospitals and non-disaster base hospitals

At the planned scale of flooding, there was no difference found in the expected rate of damaged facilities between disaster base hospitals and non-disaster base hospitals, and the expected rate of damaged beds was lower at non-disaster base hospitals (OR = 0.92, 95%CI = 0.71-1.18 for damaged facilities and OR = 0.79, 95%CI = 0.78-0.80 for damaged beds). At the maximum potential scale of flooding, there was no difference found in the expected rate of damaged facilities and beds between disaster base hospitals and non-disaster base hospitals (OR = 1.14, 95%CI = 0.95-1.38 for damaged facilities and OR = 0.99, 95%CI = 0.98-1.00 for damaged beds).

Distance between medical facilities and rivers

**Figure 12:**
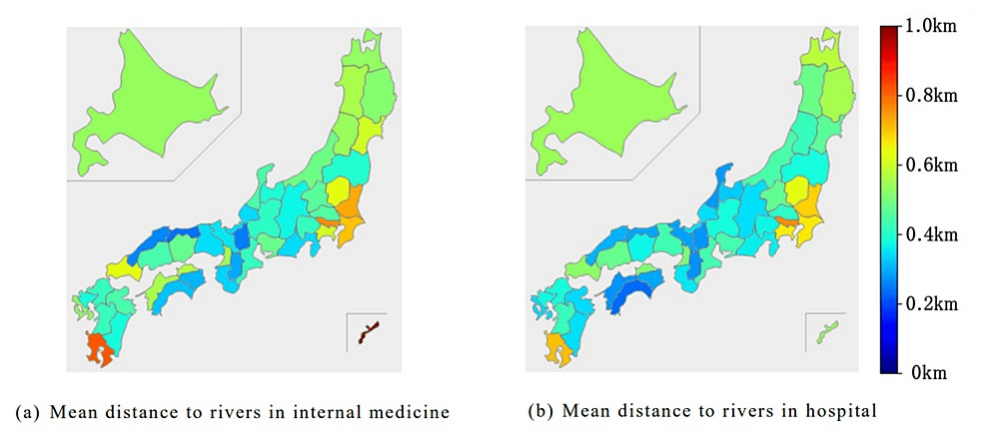
Average distance between medical facilities and the nearest river for each of the 47 prefectures

We included 56,823 internal medicine facilities and 8,126 hospitals. In several prefectures in the Kanto area, internal medicine institutions were found to be located more than 0.6 km away from rivers on average. In contrast, scattered areas in the Kinki region, the Sea of Japan side of the Chugoku region, and parts of the Shikoku region had distances less than 0.4 km. Hospitals exhibited a similar trend (Figure 12).

## Discussion

Summary of results

This study assessed the impact of floods on medical facilities registered in the Japan Medical Analysis Platform by department and region, utilizing GIS data. The planned scale of flooding is estimated to affect approximately 10% of facilities and beds across all medical departments. The maximum potential flood is estimated to affect above 20% for all departments. According to regional analysis, the Chubu and Shikoku regions were expected to suffer more severe damage. There was no difference found in the damage caused by the flood scenario between disaster and non-disaster base hospitals. These results suggest that maintaining medical services and meeting healthcare needs during a nationwide flood may be challenging.

Comparison with previous studies

Our study revealed regional variations in medical facility damage, with no specific trend toward greater impact in the Kyushu region, despite the typhoon's severity there. A previous cross-sectional study conducted in the USA revealed that hurricane damage in the USA concentrates along the East Coast, where hurricanes first make landfall [18]. This finding differs from our study. The discrepancy may arise from variations in the catchment areas of major rivers. In Japan, major rivers are susceptible to floods with equivalent rainfall because their catchment areas are smaller than those in other countries [19]. Even in the largest rivers, heavy rainfall at the headwaters can reach the river mouth within two days, causing flooding [20]. Consequently, despite regional differences in typhoon-induced precipitation in Japan, extensive floods occur in areas adjacent to rivers. In addition, Japan has a complex river system with numerous tributaries merging into the mainstream. River confluences can cause flooding due to high water volumes and complex flows [21]. Thus, the risk of floods from typhoons is high nationwide, not only in coastal regions, as our study results indicate. Also, medical facilities in the Chubu, Kinki, Chugoku, and Shikoku regions tended to be near rivers. These regions partly overlap with areas where significant damage is expected from the maximum potential scale of flooding. These results suggest that the distance between medical facilities and rivers may be related to the scale of damage.

Clinical significance

Healthcare professionals in primary care should proactively assess flood risks at their facilities and develop or integrate a business continuity plan (referred to as BCP) accordingly. We anticipate nationwide disruptions in medical facilities due to floods, which will challenge the continuity of medical care. Consequently, enhancing disaster prevention measures, both hardware and software, is imperative. Although implementing structural disaster prevention measures is challenging due to time and cost constraints, non-structural strategies have gained prominence following the Great East Japan Earthquake and the 2008 Kumamoto Earthquake, which damaged medical facilities. The MHLW has issued guidelines for developing BCP in response to floods and recommends their adoption by all medical facilities, including general clinics [22]. In fiscal year 2018, disaster base hospitals reported a 100% BCP development proportion, while non-disaster base hospitals reported approximately 25% [23]. Data on BCP adoption by general clinics in 2023 is unavailable, but reports suggest that small medical facilities face barriers such as inadequate staffing and lack of knowledge [24]. Therefore, the current BCP adoption proportion among these clinics is likely lower than that of non-disaster base hospitals. Enhancing BCP development in non-disaster base hospitals remains a critical issue, and strategies to prioritize and promote BCP adoption warrant further discussion.

We recommend that medical facilities in flood-prone areas, not limited to disaster base hospitals, prioritize the development of BCP. Medical personnel should assess the potential impact of floods in their areas and create BCP that reflect the anticipated damage. Furthermore, we expect medical personnel to actively participate in BCP development training and other programs offered by the MHLW since 2009 [23], using them as a foundation for BCP creation.

A detailed study should be conducted based on individual damage assumptions through interviews with medical facilities. These studies will assess the status of disaster response equipment, BCP formulation, and its contents. Contents include the initial response system immediately after a disaster, various manuals, and cooperation with neighboring medical facilities. In future studies, it will be necessary to interview individual medical facilities to obtain their information and add this information as a new indicator.

This study defined medical facilities expected to be flooded as damaged. However, disaster base hospitals have already implemented sufficient hardware measures in accordance with the standards of the MHLW [25]. In addition, some medical facilities have emergency facilities located on the upper floors. These medical facilities may be partially spared from damage caused by floods. In such cases, this study may be an overestimation of actual damage assumptions.

Even without direct damage, medical facilities may become inaccessible to healthcare workers and disaster victims due to blocked major highways and other barriers. Healthcare workers might not reach these facilities promptly, delaying the initial response. This situation could also result in a surge of medical needs at certain facilities, straining their operations. We must rigorously assess the indirect business impacts of flood damage and the availability of excess resources in the BCP of individual medical facilities. Moreover, future studies should include demographic data analyses to assess the equilibrium between post-flood healthcare supply constraints and increasing demand.

Strengths and limitations

To our knowledge, this study is the first comprehensive analysis of all rivers in Japan that are expected to be inundated by floods. Additionally, this study comprehensively includes nearly all insured medical institutions in Japan.

On the other hand, this study has several limitations: First, the study utilized hypothetical flood inundation area data, which may underestimate the actual extent of inundation. Consequently, the projected impact on medical facilities and beds might be greater. This point should be taken into account when interpreting this study. In addition, it is necessary to always use the latest hazard map to make damage assumptions. Furthermore, medical facilities in the inundation area are uniformly defined as damaged facilities, but in reality, the scale of damage assumed differs depending on the presence or absence of countermeasures for each facility. Second, the distance estimates assume Earth is a perfect sphere, so they may be biased. This is because the calculations use Earth's average radius of 6,371 km, ignoring the fact that Earth is actually shaped more like an ellipse. Despite this simplification, the typical error in calculating distances with this spherical assumption is usually around 0.3%, which can be considered negligible [26]. Third, the risk of inundation of medical facilities is also affected by differences in elevation, but we have not considered this aspect. We also did not take into account the extent of measures taken by individual healthcare facilities. These measures are likely to affect outcomes and require sensitivity analysis. However, additional analysis was difficult in this study due to limitations in the open data available.

## Conclusions

In Japan, floods can hinder nationwide medical functions, particularly in certain regions. Healthcare professionals should assess potential flood damage in advance and ensure that their workplace's BCP includes appropriate countermeasures.
